# Investment in family medicine to improve health outcomes in sub-Saharan Africa

**DOI:** 10.4102/phcfm.v17i1.5033

**Published:** 2025-07-28

**Authors:** Sunanda C. Ray, Mpundu Makasa, Innocent Besigye, Jacob S. Shabani, Martha Makwero

**Affiliations:** 1 Executive Committee, East Central and Southern Africa College of Family Physicians (ECSA-CFP), Arusha, United Republic of Tanzania

**Keywords:** family physicians, health system strengthening, national family physician associations, ECSA College of Family Physicians, sub-Saharan Africa

## Abstract

Family physicians (FPs), as educators, capacity builders and clinical governance leaders of primary care (PC) teams, work to make them more effective and responsive to the needs of their patients. Various strategies are required to raise the profile of Family Medicine (FM) to ensure stronger representation in health sector planning and policy development for advocacy on behalf of the communities they serve. An illustration is given of the need for FP leaders to become equal partners in the National Surgical, Obstetric and Anaesthesia Planning process to ensure safer surgery at district hospitals and to address unmet surgical needs. Integrating FM teaching throughout undergraduate medical programmes familiarises graduates with FM as a possible career choice. Collaboration with professional FP associations such as in Botswana, Kenya and Zambia has helped to define and promote the discipline of FM, increasing public and professional awareness of the specialty’s value. Promoting development of an FP scope of practice as a collaborative exercise between academic FPs and national associations assists in differentiating the roles of FPs versus non-specialist generalists. The new generation of young FPs has played a significant role in marketing FM globally, using social media platforms to support each other and to share information and best practices for managing themselves and their patients. Positioning multidisciplinary PC teams at the centre of health systems, with strong leadership from FPs, integrated people-centred care and evidence-based practices, could catalyse the intensity of change needed for more equitable, cost-effective and sustainable healthcare in Africa.

## Introduction

The Lancet Commission (2017) on the future of health in sub-Saharan Africa (SSA) recommended looking for Africa-based, home-grown solutions to address continuing health inequities, fragmented and hierarchical systems and poor health outcomes on the continent.^1^ Significant investment in strengthening existing primary care (PC) services, implemented as urban and rural facility-based care (by doctors and nurses) and community-based care (by community health workers), has also been recommended to facilitate integrated, equitable, preventive, acute and long-term care.^2^ Although PC has been promoted as the foundation of health systems since the Alma Ata Declaration in 1978, it has remained under-resourced in SSA, hindered by weak leadership, limited political commitment and perceptions of inferior status.^3^ Donor-driven vertical disease-oriented programmes are frequently planned, funded and managed centrally, operating independently of routine health services and bypassing district health leadership.^4^ We advocate that positioning multidisciplinary PC teams at the centre of health systems, with strong leadership, integrated people-centred care and evidence-based practices, could catalyse the intensity of change needed across all levels of healthcare in SSA.

Family physicians (FPs) are potentially the leaders and change agents that possess the specialist skills and insights to strengthen PC and could transform health systems.^3,5^ Unlike vertical specialists, FPs are trained to provide holistic, comprehensive care to patients, including those with complex multiple comorbidities, rather than focusing on a single disease or organ system.^6^ In most of SSA, FPs are not usually first-contact PC providers but work in district and primary hospitals or in urban health centres as leaders of clinical governance and as consultants to and educators of multidisciplinary PC teams who refer complex cases to them to manage clinically.^4,7,8^ As capacity builders, FPs work to make PC teams more effective and sustainable in achieving their strategic goals through continuing professional development (CPD) programmes, short courses, workshops and workplace training during clinical practice.^5^ By enabling PC teams to manage a wide range of conditions at PC and district levels, unnecessary specialist referrals, diagnostic testing and hospital admissions are avoided, with more cost-effective use of resources and improved availability and utilisation of services.^6,9^

## Exclusion of family physicians in health system reforms

An example of the exclusion of FP leaders from negotiations on health sector reforms can be found in discussions about the implementation of National Surgical, Obstetric and Anaesthesia Plans (NSOAPs) in African countries. These were established following the 2015 World Health Assembly Resolution 68.15 to strengthen emergency and essential surgical care as part of Universal Health Coverage.^10,11^ District hospitals in SSA are the main source of hospital care for 80% of their populations, especially those in remote and rural areas. They are often the first level of hospital providing inpatient surgery, anaesthesia and emergency care for populations of 100 000–500 000.^8,12,13^ Surgical procedures in district hospitals are mainly performed by non-specialist doctors (medical officers: MOs) or non-physician clinical officers (COs), with anaesthesia performed by COs or nurse-anaesthetists, with minimal supervision from vertical specialists. Their skills acquisition is usually through peer-to-peer instruction and experiential learning. Needs assessments noted gaps in service provision at the district level, with surgical, obstetric and anaesthesia (SOA) specialists mainly in referral hospitals.^10,11^ The NSOAPs rarely suggest how to ensure that MOs and COs should be trained and supervised to provide safer SOA services at district levels.

The African Surgical Outcomes Study (2018) followed 11 422 patients from 247 hospitals in 25 countries and revealed significant unmet surgical needs and safety concerns for patients.^14^ The number of operations per 100 000 population was 20 times lower than the crucial surgical volume required to meet a country’s essential surgical needs each year.^14,15^ The most common procedure performed was caesarean delivery, accounting for a third of cases. Patients in this study were younger with lower risk profiles and lower complication rates but were twice as likely to die as the global average in a comparative cohort of potentially avoidable causes. The authors cautioned that initiatives to improve access to surgery must be accompanied by increased resources to ensure safe surgical interventions and monitoring of patients who developed complications.^14^ A solution is to recognise FPs as educators and trainers of MOs, COs, registrars, health professional students and interns within district and PC settings, including in safer surgical practices. Their involvement in these roles and as clinical governance leaders, strengthens the overall capacity of the health workforce.^13^ Their participation promotes a culture of quality and patient safety through clinical audits, quality improvement (QI) initiatives, in-service learning opportunities and advocacy for essential equipment and technology, enabling healthcare teams to operate in safe and effective environments.^2,5^

The curriculum for training FPs to specialist Masters of Medicine (MMed) level in most of SSA includes a defined list of surgical, obstetric, emergency, trauma and anaesthetic skills appropriate for patients attending district hospitals.^9,16,17^ The list is based on a WHO Study Group report, which details procedures that should be available in district hospitals.^12^ District hospitals in SSA are small (75–250 beds) with low volumes of surgery for their catchment populations, which makes placing full-time surgical, obstetric, or anaesthetic specialists there an inefficient use of scarce resources.^13^ Authors from Canada, where FPs provide emergency, obstetric and anaesthetic services as well as PC in rural locations, advocate for generalist clinicians rather than ‘single-skilled’ specialists in rural environments because of the range of work FPs carry out in these settings while still ensuring high-quality care.^18^ In most of SSA, single-skilled specialists are reluctant to work in remote settings but could support FPs by providing outreach from referral hospitals and organising surgical camps for cataract removal, hernia repairs or children’s cleft palate surgery.^12,13^

## Advocacy for family medicine in sub-Saharan Africa: What works?

Effective advocacy occurs at multiple levels: government, academia, health institutions, international networks and communities.

### Partnerships between national associations, training institutions and governments

National FP associations represent the voice of FPs and can advocate for stronger representation of FPs on national and regional health platforms by aligning Family Medicine (FM) principles with national health priorities. Through constructive engagement over several years, Botswana and South Africa national FP associations negotiated recognition of FM by Ministries of Health and are now represented in government expert task teams and working groups on health policy development; for instance, developing position papers on health service delivery and guidelines for management of chronic diseases in PC.^19,20^ Family Medicine training is embedded in universities, legitimising FM within academic medicine and making it visible through research and teaching in undergraduate and postgraduate health professional education.^7,8,9^ Integrating FM principles throughout undergraduate medical training, taught by FPs, sensitised medical students and junior doctors to FM as a career choice, as well as creating rural training pathways with supervision and mentorship that are engaging and inspirational.^20,21,22^ Health sector research and academic publications by FPs that demonstrate improved quality of care built momentum and stakeholder confidence.^5^ Collaboration between academic FM and national associations in establishing a scope of practice for FPs helped differentiate the role of FPs versus GPs and MOs.^20^

### International networking and capacity building

The discipline of FM became better understood in Africa through advocacy by the Primary Care and Family Medicine Education Network (PRIMAFAMED), the World Organization of Family Doctors (WONCA) and other South–South collaborative partnerships between African universities.^7,9,17^ These initiatives, including partnerships with universities in high-income countries, have enabled shared learning, pooled resources, research collaborations and advocacy, which have helped establish and support FM training programmes across more than 20 African countries.^7,8,17^ There are now at least 11 countries in the East Central and Southern Africa (ECSA) region with FM training programmes and a newly established ECSA College of Family Physicians.^23,24^

### Public and professional branding

Professional FP associations such as in Botswana, Kenya and Uganda defined and promoted the identity of FM, increasing public and professional awareness of the specialty’s value.^7,20,25^ Strategic communication through media and publications is part of this branding process, through emphasising FM’s success in comprehensive, community-focused care, which serves to attract trainees and raise awareness. Young FPs and trainee FPs are leading the use of social media to network locally and internationally to provide platforms to communicate, share information and best practices in FM, promote mentorship and skills transfer and give and receive advice on managing themselves and their patients. These young professionals have been able to build strong FP identities and successfully ‘market’ FM in innovative ways that resonate with and attract young doctors to FM careers.^26^

## What has not worked well

A barrier to achieving representation in national policymaking and workforce planning bodies is a lack of awareness of the added value FPs bring to health systems compared to other generalist doctors.^5,21,25^ The confusion over what FM is and the few incentives to attract junior doctors result in poor uptake of training posts where they exist.^7,8^ Most doctors working as private general practitioners (GPs) or government MOs do not have postgraduate qualifications equipping them for this work.^3,9,17,22^ It is assumed that 1–2 years of internship at teaching hospitals prepares MOs to manage the diverse workload referred from PC to district hospitals.^22^ Medical officers are usually in district hospital posts as part of obligatory service post-qualification, which they leave on completion once they have secured postgraduate training places or to become private GPs.^8,17^ Family physicians often work in isolation and do not see themselves as FM champions or change agents. Unfortunately, the limited number of trained FPs in the region constrains their capacity to demonstrate large-scale impact.^7,8,13^

## Conclusion

### The way forward

We are at a critical stage in building resilient, people-centred health systems in SSA and need considerable support, solidarity and resources from local and international partners to take these innovations forward. Family physicians are essential to this process: their leadership, broad expertise and community focus make them key drivers of equitable health care. In response to criticisms that health services in SSA are fragmented and fragile, FPs must demonstrate that standardised training, CPD and QI audits that are integral to FM will lead to better coordination of services, improved patient care and outcomes. Conferences, campaigns, research publications and media bulletins provide opportunities for clear, consistent messaging that highlights the contribution of FPs to health systems. Partnerships between academic FM departments and national FP associations can create platforms for FPs to connect, share knowledge and collaborate across countries and disciplines, fostering professional solidarity and strengthening regional FM capacity. Scaling up training is essential to achieve visibility and influence and includes negotiating with training institutions to create more positions for FM faculty and for faculty development. Investment in young FPs, in their training, better remuneration, job security and career progression and to attract junior doctors into the specialty is essential for transforming the discipline of FM and for building a resilient PC workforce for the future.

The authors are grateful to Prof. Bob Mash and the PRIMAFAMED Network for their continued support for ECSA-CFP.

The authors S.C.R. and I.B., serve as editorial board members of this journal. S.C.R. and I.B. have no other competing interests to declare.
